# HME, NFE, and HAE-1 efflux pumps in Gram-negative bacteria: a comprehensive phylogenetic and ecological approach

**DOI:** 10.1093/ismeco/ycad018

**Published:** 2024-01-10

**Authors:** Josselin Bodilis, Olwen Simenel, Serge Michalet, Elisabeth Brothier, Thibault Meyer, Sabine Favre-Bonté, Sylvie Nazaret

**Affiliations:** Université Rouen Normandie, GlycoMEV UR 4358, SFR Normandie Végétal FED 4277, Innovation Chimie Carnot, Rouen Institute for Research and Innovation in Biomedicine, Mont-Saint-Aignan F-76821, France; Université Claude Bernard Lyon 1, UMR CNRS 5557, UMR INRAE 1418, VetAgro Sup, Ecologie Microbienne, Villeurbanne F-69622, France; Université Rouen Normandie, GlycoMEV UR 4358, SFR Normandie Végétal FED 4277, Innovation Chimie Carnot, Rouen Institute for Research and Innovation in Biomedicine, Mont-Saint-Aignan F-76821, France; Université Claude Bernard Lyon 1, UMR CNRS 5557, UMR INRAE 1418, VetAgro Sup, Ecologie Microbienne, Villeurbanne F-69622, France; Université Rouen Normandie, LMSM EA4312, Evreux F-27000, France; Université Claude Bernard Lyon 1, UMR CNRS 5557, UMR INRAE 1418, VetAgro Sup, Ecologie Microbienne, Villeurbanne F-69622, France; Université Claude Bernard Lyon 1, UMR CNRS 5557, UMR INRAE 1418, VetAgro Sup, Ecologie Microbienne, Villeurbanne F-69622, France; Université Claude Bernard Lyon 1, UMR CNRS 5557, UMR INRAE 1418, VetAgro Sup, Ecologie Microbienne, Villeurbanne F-69622, France; Université Claude Bernard Lyon 1, UMR CNRS 5557, UMR INRAE 1418, VetAgro Sup, Ecologie Microbienne, Villeurbanne F-69622, France; Université Claude Bernard Lyon 1, UMR CNRS 5557, UMR INRAE 1418, VetAgro Sup, Ecologie Microbienne, Villeurbanne F-69622, France

**Keywords:** RND, efflux pumps, antibiotic resistance, plant, metal resistance, phylogeny

## Abstract

The three primary resistance-nodulation-cell division (RND) efflux pump families (heavy metal efflux [HME], nodulation factor exporter [NFE], and hydrophobe/amphiphile efflux-1 [HAE-1]) are almost exclusively found in Gram-negative bacteria and play a major role in resistance against metals and bacterial biocides, including antibiotics. Despite their significant societal interest, their evolutionary history and environmental functions are poorly understood. Here, we conducted a comprehensive phylogenetic and ecological study of the RND permease, the subunit responsible for the substrate specificity of these efflux pumps. From 920 representative genomes of Gram-negative bacteria, we identified 6205 genes encoding RND permeases with an average of 6.7 genes per genome. The HME family, which is involved in metal resistance, corresponds to a single clade (21.8% of all RND pumps), but the HAE-1 and NFE families had overlapping distributions among clades. We propose to restrict the HAE-1 family to two phylogenetic sister clades, representing 41.8% of all RND pumps and grouping most of the RND pumps involved in multidrug resistance. Metadata associated with genomes, analyses of previously published metagenomes, and quantitative Polymerase Chain Reaction (qPCR) analyses confirmed a significant increase in genes encoding HME permeases in metal-contaminated environments. Interestingly, and possibly related to their role in root colonization, genes encoding HAE-1 permeases were particularly abundant in the rhizosphere. In addition, we found that the genes encoding these HAE-1 permeases are significantly less abundant in marine environments, whereas permeases of a new proposed HAE-4 family are predominant in the genomes of marine strains. These findings emphasize the critical role of the RND pumps in bacterial resistance and adaptation to diverse ecological niches.

## Introduction

The resistance-nodulation-cell division (RND) superfamily includes efflux pumps widely found in all living organisms. Described RND pumps catalyze substrate efflux via an H+ antiport mechanism [1]. The three primary RND families (TC #2.A.6.1 to TC #2.A.6.3 according Transporter Classification Database; TCDB) are phylogenetically close together and almost exclusively found in Gram-negative bacteria [1, 2]. Due to their wide distribution in most Gram-negative bacteria and their involvement in resistance functions against bacterial biocides, these RND pump families have been the most studied among multidrug transporters [1]. The first family (TC #2.A.6.1 or HME, for Heavy Metal Efflux) includes pumps that have been described as exclusively exporting metallic cations [3]. The second family (TC #2.A.6.2 or HAE-1, for hydrophobe/amphiphile efflux-1) includes pumps involved in the export of organic molecules toxic to bacteria, such as antibiotics, solvents, bile, detergent, or aromatic molecules [4]. Due to their very wide substrate specificity, many pumps belonging to the HAE-1 family have been implicated in the multidrug resistance (MDR) phenotype, but also in bacterial stress response and pathogenicity [5]. Efflux-mediated antibiotic resistance in Gram-negative bacteria, especially involving HAE-1 pumps, is now considered as one of the main genetic determinants for the emergence of the MDR phenotype in clinical settings [6–8]. The third family (TC #2.A.6.3 or NFE, for Nodulation Factor Exporter) includes pumps that are not yet well characterized functionally and for which the phylogenetic positioning is ambiguous, particularly with respect to the HAE-1 family. The first pump described in this family is a putative lipooligosaccharide NFE [9]. Other pumps have also been reported to be involved in the MDR phenotype [10], and one pump in this family is probably even involved in the export of Cu^2+^ [11].

In Gram-negative bacteria, RND transporters act as a tripartite complex that can bind various substrates from the periplasm and/or cytoplasm and extrude them directly to the external medium using the electrochemical potential of H+ across cell membranes. This complex is composed of a RND permease (or inner membrane protein) that is located in the cytoplasmic membrane, a periplasmic-located membrane adaptor protein that belongs to the membrane fusion protein family (MFP; TC #8.A.1), and an outer membrane channel protein (OMP; TC #1.B.17). Each of these proteins appear to be present three times (homotrimers) in the RND complex, except for some HAE-1 transporters, which have been described a heterotrimer of permeases with two different subunits [12]. The encoding genes of the RND transporter are usually organized in an operon, and the MFP and RND permease are cotranscribed, whereas in some systems, the OMP is not coorganized with the other genes such as in the case of MexXY in *Pseudomonas aeruginosa* [13].

The phylogeny of RND permeases described in the seminal work of Saier *et al*. [14] made it possible to describe the first RND families (including the HAE-1, NFE, and HME families) by attempting to link evolutionary proximity (phylogenetic clades) to functions, generally expressed by substrate specificity. Several studies have attempted to update the initial phylogeny of Saier *et al*. with both new sequences and new functional data [15–17]. However, it is often difficult to compare the phylogenetic clades and functional groups obtained from different studies, notably because of the unclear positioning of the TCDB reference sequences and the general difficulty in distinguishing the members of the HAE-1 and NFE families both phylogenetically and functionally.

At least one RND transporter is found in most Gram-negative bacteria, and a given strain generally has several RND operons [18] (http://www.membranetransport.org/). This variation in the number of pumps between and sometimes within species suggests that duplication, loss, and/or horizontal transfer of RND operon are frequent. Only a few studies have considered duplications of RND operon and the evolutionary mechanism(s) of subfunctionalization of pumps after a gene duplication [12, 19]. Other studies have shown recent horizontal gene transfers (HGTs) of genes encoding RND pumps via bacteriophage or integrative and replicative elements [20, 21]. However, the relative importance of these two evolutionary phenomena (duplication vs. HGT) has not yet been studied in RND pumps, even though it would improve the evaluation of the spread of antibiotic and metal resistance.

Based on the high prevalence and diversity of these pumps in Gram-negative bacteria, their role in antibiotic resistance might only be a secondary (byproduct) role of some HAE-1 pumps [22, 23]. Although the pumps in this family have been studied mainly in a clinical context, some data are available on their environmental substrates. For example, some HAE-1 pumps are responsible for the efflux of toxic substances of animal (bile salts) or plant origin (different phytomolecules) and may support colonization of these environments [5, 24]. A better understanding of the environmental functions of RND efflux pumps could be used to assess the selective pressures that drive the emergence of MDR phenotypes in pathogenic bacteria [23].

Our study focused on HAE1, NFE, and HME permeases in Gram-negative bacteria and aimed (i) to redefine the HAE-1, NFE, and HME families using robust phylogenetic approaches from the TCDB reference sequences and 920 genomes representative of the known diversity of Gram-negative bacteria, (ii) to compare the distribution of RND permeases in these different phylogenetic clades according to the taxonomy, origin, pathogenicity and, more generally, the ecosystem of the strains, (iii) to confirm putative ecological roles of some RND pumps both by qPCR on environmental samples using “universal” primers specifically targeting HAE-1 and HME families and by metagenome analyses, and (iv) to study the impact of HGTs and duplications on the evolutionary history of RND pumps in Gram-negative bacteria.

## Material and methods

### Genomic database and phylogenetic analyses

TCDB reference sequences of the three families HAE-1, NFE, and HME were obtained from the TCDB (http://www.tcdb.org/; in January 2021). A total of 83 protein sequences encoding an RND permease were included in the phylogenetic analysis. The 83 whole protein sequences (from 1005 to 1080 amino acids) were aligned using either Muscle 3.8.31 [25] or Clustal Omega 1.2.1 [26], with the default parameters. From each alignment, the poorly aligned positions (about two-thirds of the alignment positions) were eliminated using Gblock 0.91b [27] with the default settings. A comparison between the alignments shows that 90% of the positions (265 columns of the alignments) were found in both alignments. To introduce a minimum number of potentially misaligned positions, only the 265 positions common to both alignments were retained for subsequent analyses. Reconstruct Maximum Likelihood phylogenetic trees were obtained by using IQTree 1.6.5 [28], with a maximum parsimony starting tree. The best-fitted model (LG + F + R6) was chosen according to the Bayesian Information Criterion, and ultrafast bootstraps were determined using 1000 samples. In addition, incongruence of several polyphyletic groups was evaluated using the “tree topology test” option.

We then built an initial database from 920 reference proteomes of Gram-negative bacteria (UniProt, release 2015_09; Table S1). We excluded from our analysis the proteomes of Archaea and Gram-positive bacteria (i.e. the *Actinobacteria*, *Chloroflexi*, and *Firmicutes* phyla, except the *Negativicutes* class) and/or with an atypical wall (e.g. the *Tenericutes*, *Thermobaculum*, and *Thermotogae* phyla). A Blastp (version 2.9) search was performed in this database using each of the 83 TCDB reference sequences as a query with the default parameters, except for the e-value (<10–4) and the alignment size (>80%). Blast hits with a length of <852 amino acids, i.e. about 80% of the average size of the 83 reference sequences, were discarded (6 different sequences). A total of 6205 protein sequences were obtained, representing the putative RND permeases of the HME, HAE-1, and NFE families in 920 reference Gram-negative bacteria (Table S2). Interestingly, all the sequences identified are annotated IPR001036 and/or IPR027463 according to the protein sequence analysis and classification (InterPro version 84.0) notation, i.e. annotated as Acriflavin resistance protein and/or multidrug efflux transporter AcrB, respectively. In addition, none of these sequences matched with the reference sequences of the other RND families (families 4 to 8), using the same Blastp search parameters described above (data not shown).

The 6205 whole protein sequences (from 852 to 1637 amino acids) were aligned using Clustal Omega 1.2.1 with the default parameters. The poorly aligned positions were eliminated using Gblock 0.91b with the default settings, except parameters allowing all positions and conserved alignment blocks of at least 50 columns. A protein alignment of 650 columns was then retained for phylogenetic analyses. Reconstruct Maximum Likelihood phylogenetic trees were obtained by using IQTree 1.6.5, with a maximum parsimony starting tree. The best-fitted model (LG+F+R10) was chosen according to Bayesian Information Criterion, and ultrafast bootstraps were determined using 1000 samples.

Nine phylogenetic clades were defined in both phylogenetic trees. These clades are strongly supported with bootstrap values greater than 80% for the phylogeny obtained from 920 genomes and greater than 95% for that obtained from only the TCDB reference sequences (except for the Clade G, <21%).

From this phylogenetic analysis, we propose to define clades as RND families in Gram-negative bacteria according to the following three criteria: (i) sharing a common evolutionary origin (monophylogeny) (ii) with related functions/roles (iii) representing at least 10% of the RND pumps in the Gram-negative bacteria. We add this third conservative criterion to avoid a confusing increase in the number of families in the RND superfamily. According to these criteria, only the HME (Clade I), HAE-1 (Clades A and B), and the new HAE-4 (Clade H) families should be considered as such. Together, they account for 83.7% of the RND pumps in Gram-negative bacteria.

In addition, we propose to define a candidate status for some clades, either because their phylogenetic relationships with the three main families need to be confirmed (Clades G, C, and F) or because they represent too few sequences (Clade D). Future research should confirm or refute these last classification proposals.

### Primer design and quantitative real-time PCR assay from environmental samples

To design primer sets specific to HME and HAE-1 encoding genes, blocks of conserved amino acids were selected manually from the protein alignment described above. The primers were arranged in a set that would yield an amplicon of a size suitable for qPCR amplification (150–250 bp). Primers were designed using either several codon possibilities (i.e. degenerated primers) or the most frequent nucleotide from 1354 or 2595 nucleic sequences for HME (Clade I) and HAE-1 (Clades A and B), respectively. Their sequences were compared *in silico* with the NCBI nucleotide database version 2022/05/06 (using BLASTn 2.9) to confirm target specificity. The primer set retained to target HAE-1 family was RND_1-4_f (CAT CGT GGT GGA CGA YGC SAT YGT BRT) and RND_1-4_r (GCA CAG CAC CGG CGT VAR NGT VA), targeting the positions 1200–1450 of the RND sequence. The primer set retained to target HME family was RND_13_f (GCG GCG GTC GGY TTY ATY GCN YT) and RND_13_r (GGC CGC TGC AYY TCN GMN CC), targeting the positions 2770–3070 of the RND sequence.

qPCR amplification was performed using LightCycler 480 (Roche Diagnostics, Meylan, France) with the LightCycler® 480 software, version LCS480 1.5.0.39 (Roche Diagnostics). Twenty microliter volume contained 10 μl of LightCycler 480 SYBR Green I Master (Roche Diagnostics), 250 ng of T4 gp 32 (QBiogene), 20 pmol of each primer, and 2 ng of DNA template. The amplification conditions were as follows: 98°C for 15 min, followed by 45 cycles of 98°C for 10 s, 60°C or 65°C for 20 s (for HAE-1 or HME quantification, respectively), and 72°C for 15 s. Fluorescence was measured at the end of each cycle at 72°C, and a melting curve analysis (65–98°C) was performed at the end of the amplification procedure. For each environmental sample, two independent qPCR assays were performed from 2 ng of total DNA. The standard curve was created (in duplicate) using 10-fold dilution series of a pGEM-T Easy plasmid containing a HME or HAE-1 gene. Standard curves extend from 10 to 10^8^ copies/μl.

To check for specificity, the qPCR products from two environmental DNA samples were cloned into the pGEM-T Easy Vector System (Promega, France) according to the manufacturer’s instructions and about 50 clones were then randomly chosen for sequencing.

One hundred and sixty-eight soil samples were collected from two sites. The first site (Pierrelaye; Ile de France; France) is an agricultural area that has been amended by urban waste for two centuries. This site presents a gradient of metal contamination (0.34–7 mg kg^−1^ Cd; 39–170 mg kg^−1^ Cr; 10–342 mg kg^−1^ Cu; 21–559 mg kg^−1^ Pb; 42–1257 mg kg^−1^ Zn; 0.015–3.8 mg kg^−1^ Hg). From the second site (Lyon urban area; France), both the rhizosphere (attached to the roots of *Fallopia japonica*) and the adjacent soil (1 m from the plant) were sampled. After sampling, DNA was extracted with the MP FastDNA Spin Kit for Soil, purified, and stored at −40°C.

### Metagenome analyses

To confirm the enrichment of HAE-1 pumps in rhizosphere, we selected the study of Yeoh *et al*. [29] that compare the metagenomes of sugarcane rhizosphere and bulk soil. We chose this study because the distinction between these two compartments was clearly defined in the study, and there were enough replicates to perform robust statistical tests. We reanalyzed a total of 14 metagenomes to search for genes encoding RND permeases in Clades A and B and compared their abundance between the soil and the rhizosphere.

To investigate the link between RND pumps and saline environments, we reanalyzed nine metagenomes of sediment from different points of an estuary [30], following a salinity gradient, and compared the abundance of genes coding permeases of Clades G and H between the different points. To our knowledge, this study is one of the few conducted from an estuarine environment with enough replicates to perform statistical tests.

For these two studies, we downloaded the raw reads from Sequence Read Archive (SRA) using SRATools version 3.0.1. We assembled the forward and reverse reads and discarded reads with an average quality score <20 with bbtools version 38.96 (https://sourceforge.net/projects/bbmap/). Reads shorter than 210 base pairs were filtered with Mothur version 1.47 [31]. The remaining reads were searched with BlastX version 2.12 [32] against the in-house database of 6205 RND protein sequences with a maximum e-value of 10^−6^. Only the hits with identity percentage above 70% and alignment length above 70 amino acids were kept to reduce false positives. We then compared the relative abundance of reads coding RND permeases of interest between the different conditions. We also searched the metagenomes against the SILVA database version 138.1 to determine the abundance of 16S rRNA gene of Gram-negative bacteria in the metagenomes and calculated the number of RND reads of interest per genome of Gram-negative bacteria in each metagenome, using the Ribosome Database Project (RDP) database [33].

## Results

### Phylogeny of heavy metal efflux, nodulation factor exporter, and hydrophobe/amphiphile efflux-1 permeases

**Figure 1:**
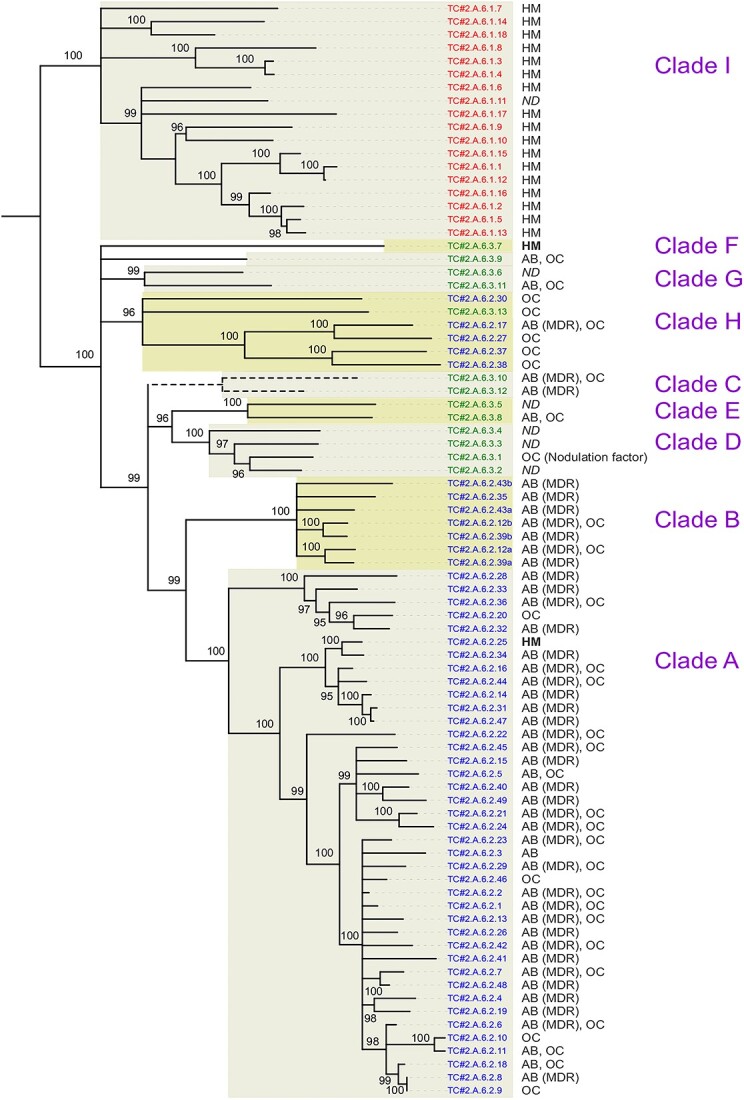
Phylogenetic tree from 83 reference RND permeases; sequences of the three families HAE-1, NFE, and HME were obtained from the Transport Classification Database (http://www.tcdb.org/; in January 2021); the color of the sequence names refers to the RND family indicated in TCDB (red for the HME-1 family, blue for HAE-1, and green for NFE); known substrates supported are indicated: a few antibiotics (AB), MDR, heavy metals (HM), organic components (OC), or not determined (ND); substrates written in bold type are outliers with respect to their position in the clades; numbers on tree branches report bootstrap results (1000 replicates); because Clade C slightly disturbs the tree by decreasing some bootstrap values, this clade was excluded for the phylogenetic reconstruction and then placed it as an indication on the tree.

**Figure 2:**
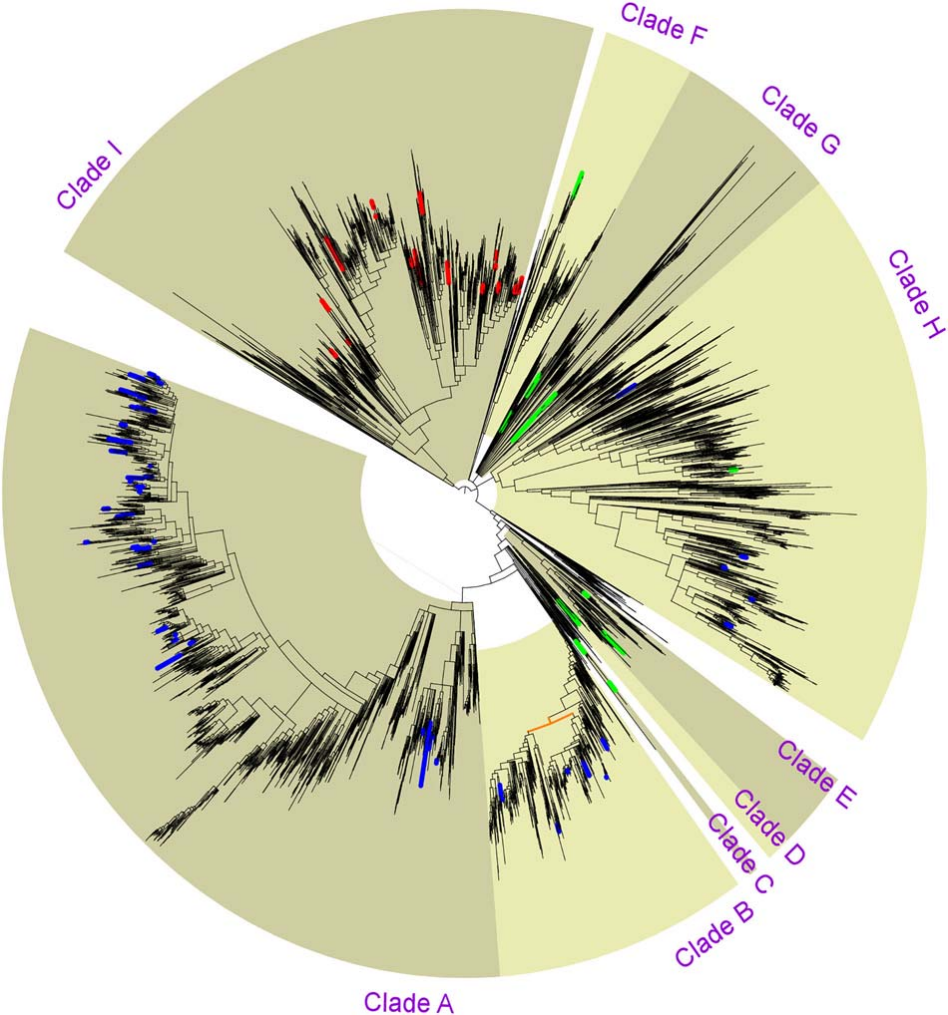
Phylogenetic tree from 6205 RND permeases; sequences of the three families HAE-1, NFE, and HME were obtained from 920 reference proteomes of Gram-negative bacteria (UniProt, release 2015_09); colored dots indicate positions of the 83 reference RND permeases described in Fig. 1; the color of the dots refers to the RND family indicated in TCDB (red for the HME-1 family, blue for HAE-1, and green for NFE); a single ancestral duplication probably occurred in Clade B (node in orange).

A first phylogenetic tree was built from 83 TCDB reference sequences of the three families HAE-1, NFE, and HME (Transport Classification Database; http://www.tcdb.org/; in January 2021) (Fig. 1). A second phylogenetic tree was built from 6205 RND sequences identified in 920 reference proteomes (UniProt, release 2015_09). The position of the TCDB reference sequences has been highlighted in this second tree (Fig. 2). The nine phylogenetic clades were defined according to the phylogeny obtained using 920 Gram-negative genomes and are reported in Fig. 1 to facilitate comparison between the two figures.

As expected, we found a monophyletic HME family (Clade I). This clade includes all the reference sequences of the HME family, i.e. the efflux pump sequences described as exporting metals, except two sequences (Fig. 1).

In contrast to the monophyletic HME family, the two HAE-1 and NFE families overlap (Fig. 1). Clade H even includes five TCDB reference sequences from the HAE-1 family and one sequence from the NFE family. For these two RND families, the hypothesis of monophyletic groups is significantly rejected (*P* < 0.01; Shimodaira-Hasegawa test). Other previous phylogenetic studies also support our results [16, 34], although the comparison between these studies is not straightforward because the TCDB reference sequences were not previously included and/or clearly identified. To facilitate comparison, the correspondences between the clades described in this study and the groups described in two other RND phylogenetic studies are presented in Table 1. Interestingly, Clades A and B include most pumps that export antibiotics belonging to several different classes (i.e. MDR phenotype). All the TCDB reference sequences of the HAE-1 family, except the five HAE-1 sequences of Clade H, are included in these two sister clades. Therefore, we propose to restrict the definition of the HAE-1 family to Clades A and B (Table 1). It can be noted that Clade C, which includes two sequences identified in the TCDB as belonging to the NFE family (TC#2.A.6.3.10 and TC#2.A.6.3.12), slightly alters the tree by decreasing some bootstrap values, particularly at the node where Clades A and B group together (93% vs. 99%; Figs S1 and S2). As this phenomenon may result from recombination between sequences, we have chosen to exclude this clade for the phylogenetic reconstruction presented in Fig. 1 and then to place it as an indication on the tree. Since the two sequences of Clade C correspond to pumps with MDR phenotype, these sequences could have diverged just after the functional subspecialization of the HAE-1 family. Because we were unable to identify a robust grouping of the three Clades A, B, and C, we define this Clade C as the only candidate HAE-1 family in Table 1.

**Table 1:** RND clades highlighted in this study.

Phylogenetic clades (in this study)	Substrates	RND family classification in TCDB	Classification proposed in this study	Proportion among RND pumps (%)	Habitats with clade proportion significantly increased	Classification proposed in Godoy *et al.*[16]	Classification proposed in Perrin *et al*. [15]
A	Antibiotics and other organic compounds	HAE-1	HAE-1	32.7	Rhizosphere	Group 1 (drugs) and Group 2	HAE-1 (Clade A)
B	Antibiotics and other organic compounds	HAE-1	HAE-1	9.1	Rhizosphere	Group 2	HAE-1 (Clade D)
C	Antibiotics and other organic compounds	NFE	Candidate HAE-1 ^a^	0.3		ND^b^	ND
D	Nodulation factors	NFE	Candidate NFE	0.8		ND	ND
E	Organic compounds and some antibiotics	NFE		2.7		ND	ND
F	Metals	NFE	Candidate HME	3.2	Metal or acid contaminated	ND	ND
G	Organic compounds and some antibiotics	NFE	Candidate HAE-4 (New family)	6.0	Marine	ND	ND
H	Organic compounds and some antibiotics	HAE-1 and NFE	HAE-4 (New family)	20.1	Marine	Group 4	HAE-1 (Clade E)
I	Metals	HME	HME	21.8	Metal or acid contaminated	Group 3 (Metals)	HME (Clade B)

a Candidate status for clades with uncertain phylogenetic relationships with the RND families (Clades C, F, and G) or with too few sequences to constitute a family.

b Not Determined. Data provided were not enough to determine the correspondence between the two classifications.

Our new analysis suggests that the NFE family is neither phylogenetically nor functionally supported. Moreover, this family was not considered in most phylogenetic studies of HAE-1 and HME permeases (Table 1). Therefore, we propose to restrict the definition of the NFE family to Clade D, which includes the only efflux pump described as exporting nodulation factors [9]. In addition, as Clade D comprises very few sequences (i.e. only 0.8% of RND pumps in the Gram-negative bacteria), we propose to define this clade as a candidate for the NFE family (Table 1).

Particular attention can also be paid to Clade B, which we propose to maintain in the HAE-1 family. This clade includes all the sequences of efflux permeases described as heterotrimer: TC#2.A.6.2.43 (a and b), TC#2.A.6.2.43 (a and b), and TC#2.A.6.2.43 (a and b), corresponding to the efflux pumps MdtBC in *Escherichia coli*, MuxBC in *P. aeruginosa*, and SmeJK in *Stenotrophomonas maltophilia*, respectively. The tandem repeats of each of these gene pairs probably result from a single ancestral duplication event highlighted in Fig. 2.

The number of genes encoding RND permeases varies greatly between the different phylogenetic clades. Clades A and B (HAE-1 family) comprise 32.7% and 9.1% of the sequences, respectively, representing 41.8% for the entire HAE-1 family. Clade I (HME family) contains 21.8% of the sequences, while Clade D (NFE family sensu stricto) contains only 0.8% of the sequences. Among the other clades, Clade H has the most sequences with 20.1% of the 6205 sequences in our study (Table 1).

Overall, the number of genes encoding RND permeases varies from 0 (72 bacterial strains) to 26 (*Spirosoma linguale* and *Singulisphaera acidiphila*), with an average of 6.7 genes per genome (Table S1). As expected, the number of genes encoding RND permeases is correlated to the size of the genome (Spearman’s rank correlation, *r* = 0.74, *P* < .01). Thus, most of the bacterial strains without RND pumps are symbiotic bacteria with a reduced genome size (43 out of 72 strains). However, some bacterial strains with a small genome have more genes encoding RND permeases than expected, e.g. *Nitrosococcus halophilus* has 10 genes encoding an RND permease with a genome size of only 1 694 969 bp, or conversely, e.g. no RND permease genes have been detected in *Deinococcus peraridilitoris* with a genome of 4 513 714 bp. Factors other than genome size are therefore also responsible for the variation in the number of copies of genes encoding RND permeases in Gram-negative bacteria.

### Environmental and physiological factors linked to resistance-nodulation-cell division phylogeny

**Figure 3:**
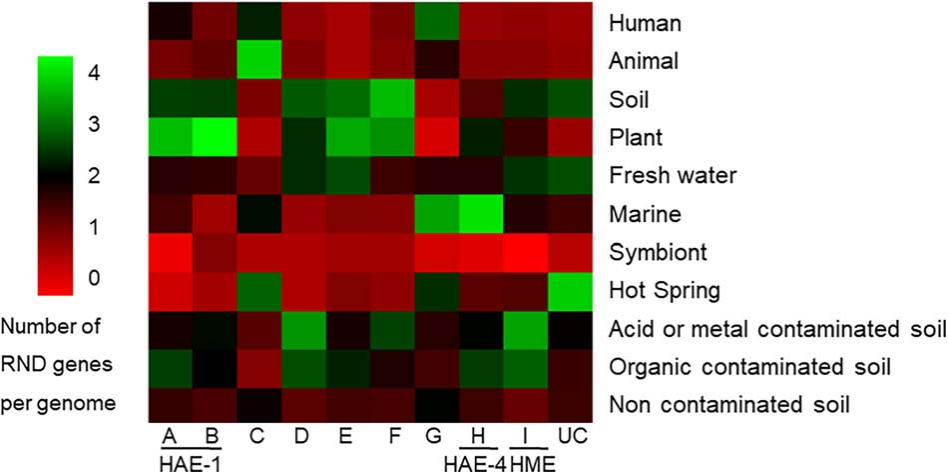
RND gene diversity in function of strain origin; heat map showing the proportion of the RND clades in function of strain habitat from our genomic database; the UC group corresponds to sequences UnClassified among the various clades described.

To better define the roles of RND pumps in the different families and clades, we then assessed links between the number of genes encoding these permeases and habitats (Fig. 3) as well as different physiological properties of the strains, such as pathogenicity or ability to nodulate (Table S1). Since there was a correlation between number of RND genes and genome size, we systematically completed statistical analyses by normalizing genome sizes and by excluding strains without RND pumps (Table S3). Table 1 presents the most significant correlations associated to RND families.

As expected, strains isolated from metal-contaminated and/or low pH habitats have more genes encoding HME permeases than strains isolated from an uncontaminated soil (*P* = 6.71 × 10^−11^; Wilcoxon test). This trend was confirmed by qPCR from environmental samples (Fig. S3).

RND pumps, and especially those of the HAE-1 family, have been primarily described in bacteria of clinical interest because of their role in MDR phenotype. In this context, we investigated a possible correlation between the pathogenicity described among our studied strains and the number or diversity of genes encoding RND permeases. Surprisingly, among the commensal bacteria in humans, pathogenic bacteria do not have more HAE-1 pumps (*P* = 0.6702; Wilcoxon test). Nevertheless, among the strains identified as pathogenic to humans, it can also be noted that opportunistic pathogens possess almost twice as many genes encoding a HAE-1 permease as compared to strict pathogens (3.3 vs. 1.8 genes per genome; *P* = 1.91 × 10^−3^; Wilcoxon test). This trend is maintained (but not statistically significant) when normalizing by genome sizes and when excluding strains without RND pumps (3.2 vs. 2.5 genes per genome; *P* = 7.47 × 10^−2^; Wilcoxon test). Moreover, strains isolated from plants have significantly more genes encoding HAE-1 permeases than strains isolated from bulk soil (Fig. 3; 6.29 vs. 4.22 copies per genomes, respectively; *P* = 1.35 × 10^−7^; Wilcoxon test). This last trend was also confirmed when normalizing by genome sizes, as well as supported by qPCR results from environmental samples (Table S3, Fig. S4). In addition, we reanalyzed a study by Yeoh *et al*. in which metagenomic data were generated from six sugarcane rhizosphere samples and eight bulk soil samples [29]. Although the proportion of genes encoding HAE1 permeases (Clades A + B) was not significantly different between the sugarcane rhizosphere and bulk soil, there was a significantly higher proportion of Clade A genes in the rhizosphere samples (Fig. S5). From the 16S rRNA genes of the Yeoh *et al*. metagenomes, we also determined the number of permeases per genome of Gram-negative bacteria. Interestingly, we found significantly more genes encoding HAE1 in the genomes of Gram-negative bacteria from the sugarcane rhizosphere as compared to the bulk soil (5.98 vs. 3.99 copies per genomes respectively; *P* = 3.33 × 10^−5^; Wilcoxon test).

Clade D (candidate NFE family) contains the only gene encoding an RND permease whose role in the export of nodulation factors has been experimentally demonstrated. Unexpectedly, among the plant-associated strains (70 strains), only 16% of the bacterial strains known to nodulate included a Clade D gene, which was slightly more than the 9% observed for those that do not nodulate (*P* = 0.1272; Wilcoxon test). Moreover, plant-strains have fewer genes encoding an RND permease of this clade compared to strains isolated from bulk soil (0.11 vs. 0.13 per genome; Fig. 3, Table S3). This last point was also supported by analysis of the metagenomes from Yeoh *et al*. (Fig.S5).

**Figure 4:**
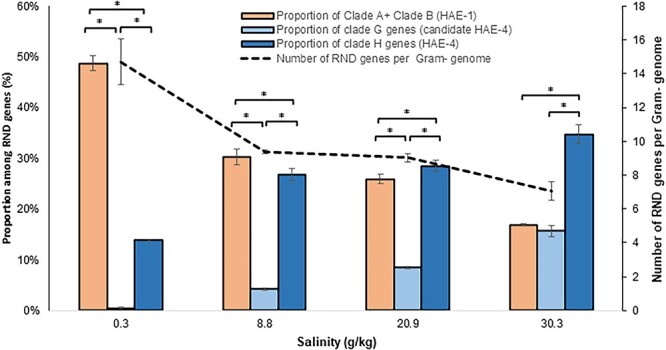
Proportion of genes encoding RND of HAE-1 (Clades A and B) and HAE-4 (Clades G and H) as a function of salinity; the dotted line indicates the number of RND permease genes per Gram-negative genome; results are indicated as mean value ± standard deviation (*n* = 3); asterisk indicates a significant difference (*P* < .05; Wilcoxon test); metagenome data from Tee *et al.* [30].

Clades G and H include 6% and 20.1% of RND sequences in our genomic database, respectively. Although the strains carrying these RND pumps are taxonomically diverse (i.e. distributed among the major phyla of our database), strains isolated from marine environments (water or sediment) have two times more genes encoding permeases in these clades, especially compared to strains isolated from fresh water and fresh sediment (Fig. 3). Moreover, in parallel with this increase in the number of genes from Clades G and H in marine environments, there is an overall decrease in the number of genes encoding RND permeases per genome and in particular a high decrease in the genes encoding the HAE-1 family of pumps (Fig. 4).

Clade F is phylogenetically close to HME family and, interestingly, as for this family, strains isolated from metal contaminated and/or low pH habitats have significantly more of these genes compared to those isolated from an uncontaminated environment (Table S3).

Clade E groups together the RND permease genes found mainly in *Cyanobacteria*. Only one of these pumps has been functionally described as exporting antibiotics (TC#2.A.6.3.8; Fig. 1). Genomic data on the habitats of origin of the strains carrying these pumps only allow us to retrieve the different ecological niches described for cyanobacteria (i.e. aquatic environments), without additional functional information.

Clade C is phylogenetically close to the HAE-1 family, with the two described RND pumps of this cluster exporting many antibiotics (Fig. 1). In our genomic database, we found only 19 RND genes in this clade (Fig. 2), which prevents assessment of correlations given physiology or environmental origin of strains.

### Origin of genetic diversity: duplication or horizontal gene transfers?

The number and diversity of genes encoding RND permeases can vary greatly between species and also, to a lesser extent, within species. Although our genomic data do not allow us to address this variation within species, 139 bacterial genera are represented by at least two genomes, with a total of 525 genomes (Table S1). Most of these genera show a variable number of genes encoding RND permeases between species (122 out of 139). For example, the *Bacteroides* genus, which is the most represented in our genomic data (31 different strains), presents from 0 to 18 genes encoding RND permeases (Table S1). Three evolutionary mechanisms can explain this variability: duplications followed by subspecialization, HGTs, and gene deletions.

Although most of the described RND permeases are associated as homotrimers, some heterotrimers have also been reported. The operons encoding these heterotrimeric pumps present a tandem repeat of two permease genes, with about 50% identity between the protein sequences [12]. From the 6205 RND proteins in our database, we systematically searched for all tandem repeats. Interestingly, 203 tandem repeats of permease genes were identified, exclusively in Clade B (HAE-1 family). The phylogenetic distribution of these gene repeats can be explained by a single ancestral tandem duplication event within this clade, followed by some deletions (Fig. 2).

Then, we investigated the impact of gene duplications (not necessarily in tandem repetition) on the variation in the number of genes coding for RND permeases between species. We used reciprocal BLAST to find genes encoding permeases with more than 95% identity within the same genome. This 95% threshold was chosen to highlight recent gene duplications and to allow easier distinction with HGTs. From our 920 reference genomes, only 8 pairs of permease genes with more than 95% identity were identified in the same genomes. The two copies of the genes are each time distant in the genome, flanking by a closed membrane fusion gene (>95% ID). Among these eight strains with putative recent duplication of RND permease genes, two belong to the same bacterial genus (*Methylobacterium*). For these two strains, a single ancestral duplication event is therefore highly probable. Moreover, for all but one of the genes involved in a duplication, elements encoding genes related to genome plasticity and/or gene transfer (transposase, integrase, phage, conjugation) were found in flanking regions (Table S4). In addition, three permease encoding genes show a percentage of GC at the third codon base (GC3) that was significantly different from the average GC3 of its original genome (Table S4). The observed duplications might result from multiple HGTs in the same strain. These putative recent RND gene duplications concern Clades A (2 duplications), I (2 duplications), E (1 duplication—before speciation event), F (1 duplication), and G (1 duplication). Although such a threshold does not allow us to clearly distinguish duplications from horizontal transfers, it is interesting to note that only 16 additional genes share between 90% and 95% identity with another gene in the same genome. Thus, for a given genome, most of the genes encoding RND permeases therefore have <90% identity with each other. Duplications of genes encoding permeases are therefore certainly too rare to explain the observed variation in the number of genes encoding RND permeases between closely related species [21, 34, 35].

We then investigated the impact of HGT on the variation between species in the number of genes encoding RND permeases. For this purpose, we also used reciprocal BLAST to search for genes encoding permeases with more than 99% identity from two strains in different genera or with more than 95% identity from two strains in different families (Table S5). This method is very conservative as it only detects recent HGTs between strains that are closely related to two of our 920 reference strains. Despite this robust but not sensitive method, we identified 15 recent horizontal transfers, i.e. two times more than the putative recent duplications of genes encoding RND permease. These HGTs only concerned Clades I (HME family; 10 HGT) and A (HAE-1 family; 5 HGT). In addition, 8 out of 15 genes involved in gene transfer display an atypical GC3 content and 6 genes are carried by a plasmid (Table S5). The same gene (i.e. >99.7% ID) was found in strains belonging to four different families. This gene was also found in two copies in *Congregibacter litoralis* (probably a “false” duplication).

To broaden the search for HGTs, we screened our RND database for the sequences with a percentage of GC at the third codon base (GC3) that was significantly different from the average GC3 of its original genome. Although this method is much more sensitive than the previous one, recent HGTs between evolutionarily distant strains but with similar GC3 were not detected. We found 604 genes encoding a permease (9.7%) with a GC3 significantly different from its original genome (Table S2). In comparison, from the same database, no sequence of the conserved *rpoB* gene (encoding sigma factor 70) displays an atypical GC3, while 33.3% (12 genes out of 36) of the genes encoding a chloramphenicol acetyltransferase detected in our reference genomes possessed this feature. HGTs would be sufficiently frequent to explain the variation in the number of genes encoding RND permeases between species in Gram-negative bacteria. These HGTs are more frequent in the HME family (14.5% of atypical GC3) than in the HAE-1 family (8.8% of atypical GC3).

Finally, we looked for the different replicons (chromosome or plasmid) carrying the genes encoding RND permeases. Among the fully assembled genomes in our database, only 3.8% of the genes encoding RND permeases are located on a plasmid, the great majority of these genes being carried by the chromosome. Furthermore, genes encoding RND permeases located on a plasmid do not present an atypical GC3 more often than those located on the chromosome (9.4% vs. 9.9%, respectively; *P* = 1 Chi^2^ test). Therefore, HGTs essentially involve genes encoding RND permeases carried by chromosomes.

## Discussion

The first phylogenetic study analyzing the members of the RND superfamily revealed that these proteins fall into seven families that cluster on the phylogenetic tree in accordance with function [36], and an eighth family was later described and added to the RND superfamily [37]. In this study, we focused on the three primary RND families, which are phylogenetically related, almost exclusively found in gram-negative bacteria, and involved in resistance against bacterial biocides and metals [1, 2]. The main objective of this study was to better characterize these three families phylogenetically and to attempt to link the diversity of these pumps to their function by using an original ecological approach.

### Heavy metal efflux family

Our phylogenetic study, including the reference RND permeases from the TCDB database, confirms the monophyly of the HME family (Clade I), which is thus well-circumscribed phylogenetically and functionally. Only two pumps previously characterized as carrying metals are found in other clades. GesABC (TC#2.A.6.2.25), which is found in Clade A (HAE-1 family), is induced in the presence of gold (Au+) and provides resistance to this metal [38]. However, this pump exports many organic compounds, including antibiotics, without evidence of direct Au^+^ uptake [38, 39]. This pump should therefore probably remain classified in the HAE-1 family. The second RND efflux pump described as exporting metals but evolutionarily distant from other HME pumps is Cus3ABC (TC#2.A.6.3.7). This pump is induced and likely transports copper (Cu^2+^), with no organic substrate described at this time [11]. Although Moraleda-Muñoz *et al*. had classified this efflux pump in the HME family, it belongs to the NFE family according to the TCDB classification [11]. Since we have shown in our study that the NFE family has no phylogenetic or functional consistency, we found this atypical pump in a particular clade (Clade F) evolutionarily close to Clade I (HME family *sensu stricto*). In fact, metadata associated with our 920 representative genomes, as well as analyses of previously published metagenomes and quantitative qPCR analyses, confirmed a significant enrichment of genes from Clade I but also Clade F, in acid and metal-contaminated environments. Although phylogenetic analysis failed to define Clades I and F as sister clades, we propose to define Clade F as a candidate for the HME family (Table 1).

Some authors have described subfamilies within the HME family, specialized for specific metals [3]. Although we were unable to address the level of resolution of these studies, it did confirm subspecializations within the HME pumps. We systematically found several different genes encoding HME permeases in the genomes of strains adapted to high metal concentrations (2.8 on average with a maximum of 12 genes for *Methylobacterium extorquens*). The two closest sequences among the 12 sequences encoding an HME permease in *M. extorquens* present only 91.08% identity between them. If all of the HME pumps were functionally equivalent, a modification of the regulation of an operon encoding an HME pump or even an amplification of the number of copies of a same gene would have been evolutionarily easier and sufficient to increase the adaptation of bacteria to these contaminated environments. However, our study related the role of HGTs of various HME genes to the adaptation of strains to metal contamination, with Clade I being the most affected by HGTs among the RND pumps. The number of RND permease genes, and probably also their diversity, should represent a relevant bioindicator to study the impact of metal pollution. The new primers designed in our study for detecting all the genes of this family could be useful to screen different samples.

Considering all these observations, we hypothesized that metal export by RND pumps is a substrate specificity that appears very rarely during evolution. A single evolutionary event before the common ancestor of Clades I and F could be enough to explain the phylogeny of HME permeases. We also argue that substrate specificity is exclusive between metals and organic molecules, evolutionarily irreversible with respect to metals. This substrate specificity is important for understanding the coselection of antibiotic resistance in metal-contaminated environments. Thus, an abundance of antibiotic resistance genes has been reported in metal-polluted environments, mostly of these genes encoding efflux permeases [40, 41]. These coselection mechanisms may include coresistance (different resistance determinants present on the same genetic element), cross-resistance (the same genetic determinant responsible for resistance to antibiotics and metals), and coregulation [42]. Although mechanisms of coresistance and coregulation of resistance to antibiotics and metals are extensively described in the literature [43], our study suggests that poor annotation of ARG databases due to the current ambiguous classification of RND pumps may be responsible for overestimating cross-resistances in metagenome studies. Furthermore, most of the described HAE-1 pumps carry a wide variety of substrates, including organic contaminants. Among these RND pumps, some have been described to efflux both polycyclic aromatic hydrocarbons (PAHs) and antibiotics [44]. In addition, bacterial isolates tolerant to PAHs and/or capable of degrading PAHs from highly contaminated environments often exhibit strong resistance to metals and antibiotics [45, 46]. We suggest that the reported coselection between antibiotics and metal resistances could also arise from multicontamination (e.g. metals and PAHs) of most of the sites studied [45, 47].

### Hydrophobe/amphiphile efflux-1 family

Our phylogenetic study including the reference HAE-1 permeases from the TCDB database was used to propose a more phylogenetically and functionally consistent definition of this family. Thus, we propose to restrict the HAE-1 family to Clades A and B, including most pumps that export antibiotics belonging to several classes (i.e. involved in MDR phenotype). Moreover, a single ancestral tandem duplication event within Clade B, followed by a few deletions, could explain the tandem repeats of permease genes exclusively found in this clade that resulted in a heterotrimer RND pump. This ancestral duplication probably occurred in *Proteobacteria*, before the split into Alpha-, Beta-, and Gamma-classes [12].

RND pumps, and especially those of the HAE-1 family, have been primarily described in bacteria of clinical interest because of their role in conferring MDR phenotype. In this context, we investigated a possible correlation between the pathogenicity described among our studied strains and the number and diversity of genes encoding RND permeases. Although the genes encoding RND permeases, especially in HAE-1 family, are mostly carried on chromosomes, we have shown that they are quite often transferred laterally, so that HGT is the major evolutionary force behind copy number variations between and even within species. Nevertheless, we did not find an enrichment of genes encoding HAE-1 permeases in human pathogens as compared to nonpathogenic commensal bacteria. In addition, opportunistic pathogens whose primary habitat is environmental have a significantly higher number of genes encoding HAE-1 permeases compared to strict pathogens. The selective pressure of intensive antibiotic use thus seems too recent and sporadic to result in significant enrichment of HAE-1 permeases among commensal strains including human pathogens.

In contrast, we found a very significant increase in the number of genes encoding HAE-1 permeases in strains isolated from the rhizosphere, compared to all other environments, including bulk soil. Plants produce exudates composed of many molecules used as substrates by the microbiota (sugars, amino acids, vitamins) but also secondary metabolites (e.g. flavonoids, benzoxazines, shikonin) some of which are known to be involved in resistance to plant pathogens [48, 49]. The rhizosphere harbors a very high density and diversity of microbial species due to plant exudation of nutrients. Rhizospheric bacteria are therefore subject to relatively high competition with other microorganisms, some of which secrete antibacterial compounds. They must also contend with a cocktail of toxic phytomolecules. The number and the diversity of HAE-1 pumps probably plays a central role in this colonization of the rhizosphere, by allowing bacteria to balance efflux of various toxic molecules with metabolic leaks.

In addition, several opportunistic pathogens are enriched in the rhizosphere, such as some species belonging to the genera *Achromobacter*, *Burkholderia*, *Enterobacter*, *Herbaspirillum*, *Ochrobactrum*, *Pseudomonas*, *Ralstonia*, *Staphylococcus*, and *Stenotrophomonas* [50–53]. These bacterial genera are characterized by a large number of HAE-1 genes, high intrinsic antibiotic resistance, and the potential to evolve to an MDR phenotype in clinical settings. Plant–bacteria relationships are much older than the clinical use of antibiotics. Hundreds of millions of years of coevolution between plants and their microbiota have probably mediated this preadaptation of HAE-1 pumps to the intensive use of antibiotics, of natural and even synthetic origin, particularly in opportunistic rhizospheric pathogenic bacteria. Searching for natural inhibitors of RND pumps in plant roots likely has a high potential to discover molecules that may counter the emergence of MDR strains in clinical settings [54].

### New hydrophobe/amphiphile efflux-4 family

Although we have shown in our study that the NFE family has no phylogenetic or functional consistency, two clades evolutionary close to each other (Clades H and G) could correspond to a new RND pump family. These two clades represent 26.1% of the RND sequences in our genomic database and are distributed across the Bacteria domain. Although pumps of the HAE-1 family are less abundant in marine environments, pumps of these clades become predominant among RND pumps (Fig. 4). The nine functionally characterized pumps of these clades are responsible for the efflux of organic molecules, including antibiotics for at least three of them (Fig. 1). A Clade H pump (VexEF; TC#2.A.6.2.30) requires Na^+^ for drug extrusion from cells [55]. The pumps of Clades G and H are globally more abundant in environments with high salinity, and thus, this functional feature may be common to all pumps of this proposed new family. A role of these pumps could be to manage osmotic pressure. This hypothesis should be tested and new investigations initiated to determine the precise role of Na^+^ in the function of these RND pumps. The substrates of this new putative family being organic molecules, we propose to name it the HAE-4 family. Three other HAE families already exist within the RND superfamily. According to our phylogenetic analysis, however, Clades G and H do not form a robust monophyletic group (Figs 1 and 2). We propose to restrict the new family to Clade H alone (20.1% of the RND sequences in Gram-negative bacteria) and to define Clade G as a candidate for the HAE-4 family (Table 1).

In summary, this work has clarified the phylogeny and more generally the classification of the three primary RND families in Gram-negative bacteria, using an ecological approach. The identification of the HME and HAE-1 families has been clarified with a positioning of reference sequences that should facilitate the practical definition of these families and the comparison between different studies, especially with untargeted metagenome data. Moreover, although the NFE family should no longer be considered as an RND family, we propose to replace it by the HAE-4 family, which is more phylogenetically delimited and seems to present new functional specificities. Finally, for each of these RND families, we have highlighted a major role in the adaptation of bacteria to their ecological niches.

## Supplementary Material

Supplemental_Figures_ycad018

Table_S1_List_of_strains_genomic_database_ycad018

Table_S2_List_of_RND_permease_genomic_database_ycad018

Table_S3_statistical_analyses_in_genomic_database_ycad018

Table_S4_putative_gene_duplication_genomic_database_ycad018

Table_S5_putatitve_HGT_genomic_database_ycad018

## Data Availability

All data reported in this paper will be shared by the lead contact upon request.
