# Supplementary material for: HME, NFE, and HAE-1 efflux pumps in Gram-negative bacteria: a comprehensive phylogenetic and ecological approach
Source: ISME Commun. 2024 Jan 10;4(1):ycad018. doi: 10.1093/ismeco/ycad018 (PMC10872679; doi:10.1093/ismeco/ycad018)
Supplement: Supplemental_Figures_ycad018 [file supplemental_figures_ycad018.docx]

**Supplemental figures**

HME, NFE and HAE-1 efflux pumps in Gram-negative bacteria: a comprehensive phylogenetically and ecological approach

Josselin Bodilis^1,2^, Olwen Simenel^1,2,3^, Serge Michalet^2^, Elisabeth Brothier^2^, Thibault Meyer^2^, Sabine Favre-Bonté^2^, and Sylvie Nazaret^2^

^1^ Université Rouen Normandie, GLYCOMEV UR 4358, SFR Normandie Végétal FED 4277, Innovation Chimie Carnot, IRIB, F-76000 Rouen, France

^2^ Université de Lyon, Université Claude Bernard Lyon 1, UMR CNRS 5557, UMR INRAE 1418, VetAgro Sup, Ecologie Microbienne, F-69622 Villeurbanne, France.

^3^ Université Rouen Normandie, LMSM EA4312, F-27000 Evreux, France.

**Figure S1.** Phylogenetic tree from 83 reference RND permeases (including the two sequences of the Clade C). Clade C (TC#2.A.6.3.10 and TC#2.A.6.3.12) slightly disturbs the tree by decreasing some bootstrap values (e.g., 93% vs 99% for the node grouping Clades A and B). This clade was excluded for the phylogenetic reconstruction presented in Figure 1 (see also Fig. S2) and then placed as an indication on the tree.

**Figure S2.** Phylogenetic tree from 81 reference RND permeases (excluding the two sequences of the Clade C; TC#2.A.6.3.10 and TC#2.A.6.3.12). Compared with the tree in Figure 1, nodes with bootstraps < 95% are not collapsed and Clade C is not added.


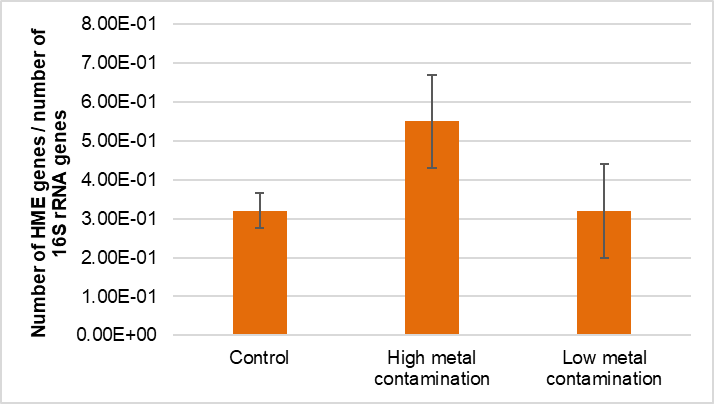


b

a

a

**Figure S3.** Relative abundance of genes encoding HME permease in bacterial soil communities from control (n=9), high (n=35)- and low-metal-contaminated soils (n=12). The site (Pierrelaye; Ile de France; France) is an agricultural area that has been amended by urban waste for two centuries. Quantification of the numbers of gene was realized by qPCR analyses. Different letters indicate a significant difference between proportion of genes encoding HME permease (P<0.05; Wilcoxon test with post hoc testing).

**Figure S4.** Relative abundance of genes encoding HAE-1 pumps in bacterial soil communities from 14 urban sites of the Lyon area (Auvergne-Rhône-Alpes, France; n=4). Quantification of the numbers of gene was realized by qPCR analyses. From these 14 paired samples of *Fallopia japonica* rhizosphere and adjacent bulk soil taken together, a significant higher abundance of genes encoding HAE-1 was found in rhizosphere compared to bulk soil (P<0.05; t test).

*

*

*

*

*

*

*

*

*

**Figure S5.** Proportion of permease genes of different clades in the sugarcane rhizosphere (n=6) and bulk soil (n=8), from metagenomic data in Yeo et al [28]. Asterix indicates a significant difference (P<0.05; Wilcoxon test).
